# Poliovirus surveillance in patients with primary immunodeficiencies, India

**DOI:** 10.2471/BLT.22.289066

**Published:** 2023-05-01

**Authors:** Madhu Chhanda Mohanty, Ahmad Mohammad, Harish Verma, Arun Kumar, Manisha Ranjan Madkaikar, Mukesh Desai, Swapnil Yashwant Varose, Unnati Sawant, Reetika Malik Yadav, Prasad Taur, Rachana Kathuria, Manogat Tatkare, Megh Murhekar, Pradeep Haldar, Priya Abraham

**Affiliations:** a National Institute of Virology Mumbai Unit, Indian Council of Medical Research, Haffkine Institute Campus, Acharya Donde Marg, Parel, Mumbai-400012, India.; b World Health Organization Country Office, New Delhi, India.; c Polio Eradication, World Health Organization, Geneva, Switzerland.; d National Institute of Immunohaematology, Indian Council of Medical Research, Mumbai, India.; e Bai Jerbai Wadia Hospital for Children, Mumbai, India.; f Ministry of Health and Family Welfare, New Delhi, India.; g National Institute of Virology, Indian Council of Medical Research, Pune, India.

## Abstract

Individuals with primary immunodeficiencies who are infected with vaccine-derived polioviruses may continue to shed poliovirus for months and go undetected by surveillance programmes of acute flaccid paralysis. These patients therefore pose a risk of initiating poliovirus outbreaks that jeopardize efforts towards global polio eradication. To identify these individuals, we designed a study protocol for the establishment of a network for surveillance of immunodeficiency-related vaccine-derived poliovirus in India. In the first step we identified recognized centres in India that could diagnose and enrol patients with primary immunodeficiency disorder into the study. Stool sample collection from study sites, culture, isolation, characterization of enteroviruses and reporting to study sites was carried out at the National Institute of Virology Mumbai Unit, as per the WHO national polio surveillance project protocol. In the first phase of the study from January 2020 to December 2021, we implemented the protocol at seven study sites at different medical institutes to determine the proportion of poliovirus infections in primary immunodeficiency disorder patients of India. We later expanded the study by including an additional 14 medical institutes across the country in the second phase running from January 2022 to December 2023. We believe this study protocol will help other countries to initiate immunodeficiency-related vaccine-derived poliovirus surveillance to identify and follow up patients who are long-term excretors of vaccine-derived poliovirus. Integration of immunodeficiency-related poliovirus surveillance with acute flaccid paralysis surveillance of the existing poliovirus network will enhance continuous screening of patients with primary immunodeficiency disorder in the future.

## Résumé

Certains individus qui présentent des immunodéficiences primaires et sont infectés par des poliovirus dérivés d'une souche vaccinale pourraient continuer à excréter le poliovirus pendant des mois sans que ce dernier ne soit détecté par le biais d'une surveillance de la paralysie flasque aiguë. Ces patients risquent donc de déclencher des épidémies de poliovirus qui mettent en péril les efforts visant à éradiquer la poliomyélite dans le monde. En vue d'identifier ces individus, nous avons élaboré un protocole d'étude pour établir, en Inde, un réseau de surveillance du poliovirus d'origine vaccinale lié à une immunodéficience. Au cours de la première étape, nous avons repéré des centres reconnus dans le pays, capables de diagnostiquer des patients atteints d'un syndrome d'immunodéficience primaire et de les recruter dans le cadre de l'étude. Le prélèvement des échantillons de selles auprès des sites participant à l'étude, la culture, l'isolement, la caractérisation des entérovirus et la communication des résultats à ces sites ont été pris en charge par le National Institute of Virology Mumbai Unit, conformément au protocole du Projet national de surveillance de la poliomyélite de l'OMS. Nous avons consacré la première phase de l'étude, qui s'est déroulée entre janvier 2020 et décembre 2021, à la mise en œuvre du protocole au sein de différents établissements médicaux sur sept sites participants, afin de déterminer le nombre d'infections au poliovirus chez les patients souffrant d'un syndrome d'immunodéficience primaire en Inde. Nous avons ensuite, durant la deuxième phase comprise entre janvier 2022 et décembre 2023, élargi l'étude en incluant 14 établissements supplémentaires à travers le pays. Nous sommes convaincus que ce protocole d'étude aidera d'autres pays à instaurer une surveillance du poliovirus dérivé d'une souche vaccinale et lié à une immunodéficience, qui leur servira à identifier et suivre les patients responsables d'une excrétion prolongée du poliovirus d'origine vaccinale. L'intégration, au sein du réseau existant dédié au poliovirus, d'une surveillance de ce type couplée à une surveillance de la paralysie flasque aiguë améliorera le dépistage systématique des patients atteints d'un syndrome d'immunodéficience primaire à l'avenir.

## Resumen

Las personas con inmunodeficiencias primarias infectadas por los poliovirus de origen vacunal pueden seguir excretando poliovirus durante meses sin que la vigilancia de la parálisis flácida aguda los detecte. Por lo tanto, estos pacientes suponen un riesgo de iniciar brotes de poliovirus que pongan en peligro los esfuerzos hacia la erradicación mundial de la poliomielitis. Para identificar a estas personas, diseñamos un protocolo de estudio para el establecimiento de una red de vigilancia de poliovirus de origen vacunal relacionados con inmunodeficiencias en la India. En el primer paso identificamos centros reconocidos en la India que pudieran diagnosticar e inscribir en el estudio a pacientes con trastorno de inmunodeficiencia primaria. La recogida de muestras de heces de los centros de estudio, el cultivo, el aislamiento, la caracterización de los enterovirus y la notificación a los centros de estudio se llevaron a cabo en el Instituto Nacional de Virología, Unidad de Mumbai, según el protocolo del Proyecto Nacional de Vigilancia de la Poliomielitis de la OMS. En la primera fase del estudio, de enero de 2020 a diciembre de 2021, aplicamos el protocolo en siete centros de estudio de diferentes institutos médicos para determinar la proporción de infecciones por poliovirus en pacientes con trastorno de inmunodeficiencia primaria de la India. A continuación, ampliamos el estudio con la inclusión de otros 14 institutos médicos de todo el país en la segunda fase, de enero de 2022 a diciembre de 2023. Creemos que este protocolo de estudio ayudará a otros países a iniciar la vigilancia de poliovirus de origen vacunal relacionados con la inmunodeficiencia para identificar y hacer un seguimiento de los pacientes que son excretores a largo plazo de poliovirus de origen vacunal. La integración de la vigilancia del poliovirus asociado a la inmunodeficiencia con la vigilancia de la parálisis flácida aguda de la red de poliovirus existente mejorará el cribado continuo de pacientes con trastorno por inmunodeficiencia primaria en el futuro.

## ملخص

إن الأفراد الذين يعانون من نقص المناعة الأولية، المصابين بفيروس شلل الأطفال المشتق من اللقاح، يمكنهم الاستمرار في التخلص من فيروس شلل الأطفال على مدار عدة أشهر، ولا يتم اكتشافهم من خلال مراقبة الشلل الرخو الحاد. وبالتالي، فإن هؤلاء المرضى يشكلون خطرًا لبدء حالات تفشي فيروس شلل الأطفال، والتي من شأنها أن تعرض الجهود المبذولة لاستئصال شلل الأطفال عالميًا للخطر. لتحديد هؤلاء الأفراد، قمنا بتصميم بروتوكول دراسة لإنشاء شبكة لمراقبة فيروس شلل الأطفال المرتبط بنقص المناعة، والمشتق من اللقاح في الهند. في الخطوة الأولى، قمنا بتحديد مراكز معروفة في الهند يمكنها تشخيص وتسجيل المرضى المصابين باضطراب نقص المناعة الأولية في الدراسة. تم جمع عينات البراز من مواقع الدراسة والانتشار والعزل، وتوصيف الفيروسات المعوية، وتقديم التقارير إلى مواقع الدراسة في المعهد الوطني للفيروسات، وذلك عن طريق وحدة مومباي وفقًا لبروتوكول المشروع الوطني لمراقبة شلل الأطفال التابع لمنظمة الصحة العالمية. في المرحلة الأولى من الدراسة من يناير/كانون الثاني 2020 إلى ديسمبر/كانون الأول 2021، طبقنا البروتوكول في سبعة مواقع للدراسة في معاهد طبية مختلفة لتحديد نسبة حالات عدوى فيروس شلل الأطفال في مرضى اضطراب نقص المناعة الأولية في الهند. فيما بعد، قمنا بتوسيع الدراسة من خلال تضمين 14 معهدًا طبيًا إضافيًا في جميع أنحاء الدولة في المرحلة الثانية الممتدة من يناير/كانون الثاني 2022 إلى ديسمبر/كانون الأول 2023. نحن نعتقد أن هذا البروتوكول للدراسة سوف يساعد الدول الأخرى على تدشين مراقبة فيروس شلل الأطفال المشتق من اللقاح، المتعلق بنقص المناعة، لتحديد ومتابعة المرضى الذين يظهر لديهم فيروس شلل الأطفال المشتق من اللقاح على المدى البعيد. إن تكامل مراقبة فيروس شلل الأطفال المرتبط بنقص المناعة، مع مراقبة الشلل الرخو الحاد في شبكة فيروس شلل الأطفال الحالية، سوف يعزز من الفحص المستمر للمرضى المصابين باضطراب نقص المناعة الأولية في المستقبل.

## 摘要

感染了疫苗衍生脊髓灰质炎病毒的原发性免疫缺陷患者可能继续传播脊髓灰质炎病毒数月而未在急性弛缓性麻痹监测中被发现。因此，这些患者有可能导致脊髓灰质炎病毒的爆发，从而影响全球根除脊髓灰质炎行动。为了识别这些患者，我们设计了一个研究方案，即在印度建立一个与免疫缺陷相关的疫苗衍生脊髓灰质炎病毒监测网络。在第一步中，我们确定了印度公认的可以诊断原发性免疫缺陷病并将患者纳入研究的中心。孟买国家病毒学研究所根据《世卫组织国家脊髓灰质炎监测项目》方案，从研究场所采集粪便样本，进行肠道病毒的培养、分离、鉴定并向研究场所报告。在 2020 年 1 月至 2021 年 12 月的第一阶段研究中，我们在不同医疗机构的 7 个研究场所实施了该方案，以确定印度原发性免疫缺陷病患者中脊髓灰质炎病毒感染的比例。后来，我们扩大了研究的范围，在 2022 年 1 月至 2023 年 12月 的第二阶段研究中，纳入了全国另外 14 家医疗机构。我们相信，该研究方案将有助于其他国家实施与免疫缺陷相关的疫苗衍生脊髓灰质炎病毒监测，以识别和随访长期携带和排出疫苗衍生脊髓灰质炎病毒的患者。将与免疫缺陷相关的脊髓灰质炎病毒监测与现有脊髓灰质炎病毒网络中的急性弛缓性麻痹监测相结合，将有助于加强未来对原发性免疫缺陷并患者的持续筛查。

## Резюме

Лица с первичными иммунодефицитами, инфицированные полиовирусами вакцинного происхождения, могут продолжать выделять полиовирус в течение нескольких месяцев и оставаться невыявленными в ходе эпиднадзора за острым вялым параличом. Поэтому эти пациенты представляют риск возникновения вспышек полиовируса, которые ставят под угрозу усилия по ликвидации полиомиелита в мире. Для выявления таких лиц был разработан протокол исследования для создания сети эпиднадзора за полиовирусом вакцинного происхождения, связанным с иммунодефицитом, в Индии. На первом этапе были определены признанные центры в Индии, которые могли диагностировать и включить в исследование пациентов с первичным иммунодефицитом. Сбор образцов кала в исследовательских центрах, культивирование, выделение, характеристика энтеровирусов и предоставление отчетов в исследовательские центры проводились в Национальном институте вирусологии, подразделение Мумбаи, в соответствии с протоколом Национального проекта ВОЗ по эпиднадзору за полиомиелитом. На первом этапе исследования с января 2020 года по декабрь 2021 года протокол был внедрен в семи исследовательских центрах при различных медицинских учреждениях для определения доли полиовирусных инфекций у пациентов с первичным иммунодефицитом в Индии. В дальнейшем исследование было расширено за счет включения еще 14 медицинских учреждений по всей стране на втором этапе, который продлится с января 2022 года по декабрь 2023 года. Считается, что данный протокол исследования поможет другим странам начать эпиднадзор за полиовирусом вакцинного происхождения, связанным с иммунодефицитом, с целью выявления и последующего наблюдения за пациентами, длительно выделяющими полиовирус вакцинного происхождения. Интеграция эпиднадзора за полиовирусом, связанным с иммунодефицитом, с эпиднадзором за острым вялым параличом в рамках существующей сети по борьбе с полиовирусом обеспечит непрерывный скрининг пациентов с первичным иммунодефицитом в будущем.

## Introduction

In late 2022, a resurgence of paralytic polio cases from vaccine-derived poliovirus was detected in Israel and the United States of America (specifically, New York).^1^ In the United Kingdom of Great Britain and Northern Ireland, since February 2022, the World Health Organization (WHO) Global Polio Laboratory Network has consistently detected Sabin-like type 2 poliovirus isolates in sewage samples collected from London.^2^ Without global eradication, there is a risk of reinfection from importation and spread of wild poliovirus or vaccine-derived poliovirus, or new emergence and circulation of vaccine-derived poliovirus. 

Vaccine-derived polioviruses are defined as viruses demonstrating a genetic divergence of > 1% nucleotide changes from the parent Sabin strain for types 1 and 3, or > 0.6% nucleotide changes for type 2 in the VP1 region of the nuceleotide.^3^ Oral polio vaccine has the potential to revert to a neurovirulent form if it persists for a long period in immunodeficient persons or circulates in under-vaccinated communities. Unlike immunocompetent persons, who excrete the vaccine virus for a limited period, some immunodeficient persons are unable to clear intestinal replication of the vaccine virus after exposure to oral polio vaccine. Immunodeficiency-related vaccine-derived polioviruses therefore pose a threat to the polio eradication campaign, as individuals that harbour the virus for prolonged periods of time could serve as sources of poliovirus reintroduction after polio eradication.^4^^,^^5^ These people could also go undetected by acute flaccid paralysis surveillance for the detection of polio cases, as the immunodeficient person may not be affected by paralysis.^6^

Primary immunodeficiency disorders are genetic disorders in individuals who have an incomplete immune system.^7^ There are over 450 different types of known primary immunodeficiency disorders identified to date.^8^ Often thought to be rare, primary immunodeficiency disorder occurs as frequently as 1 in 2000 children, 1 in 1200 people of all ages, and 1 in 600 households, according to a population-based survey in the USA.^9^^–^^11^

Despite advancements in the diagnosis of immunodeficiency, the diagnosis and care of primary immunodeficiency disorders in India is still developing and requires better awareness and infrastructure if care is to be available countrywide. To overcome these challenges, the Indian Council of Medical Research has established the Centre for Advanced Research for Primary Immunodeficiency Disorders to train scientists and physicians, and to establish standardized diagnostic techniques and facilities.^10^ Other organizations have also contributed towards raising awareness of the issue and helping affected people to access proper care.^11^^–^^14^

Current poliovirus surveillance in India is based on acute flaccid paralysis surveillance which can detect transmission of poliovirus by patients with paralysis. However, patients with primary immunodeficiency disorder not affected by paralysis may continue to shed vaccine-derived poliovirus for months and go undetected by acute flaccid paralysis surveillance. There is a risk that these patients can cause spillover circulation of poliovirus in the community, leading to circulating vaccine-derived poliovirus. This concern led the Strategic Advisory Group of Experts on Immunization and the Global Polio Eradication Initiative to recommend expanding poliovirus surveillance among people with primary immunodeficiency disorder, as a strategy for the period after certification of polio eradication.^15^ WHO has supported several pilot studies in various countries in efforts to establish immunodeficiency-related vaccine-derived poliovirus surveillance.^16^

To address the issue, the Indian Council of Medical Research led a study to investigate poliovirus infection in children with primary immunodeficiency disorders.^17^ The study was a collaboration among the National Institute of Virology in Mumbai (a global specialized laboratory for polio), the National Institute of Immunohaematology in Mumbai (a centre of excellence for the diagnosis of primary immunodeficiency disorders) and Bai Jerbai Wadia Hospital for Children in Mumbai (a tertiary care unit for children). The study identified several children with primary immunodeficiencies with polio and non-polio enterovirus infections, as well as one child who was a long-term excretor of vaccine-derived poliovirus type 3. This patient had severe combined immunodeficiency with 10% nucleotide change from the parent Sabin strain during 2 years of immunodeficiency-related vaccine-derived poliovirus type 3 excretion. The study helped to build support for implementing screening for long-term poliovirus excretors in India.^18^

We therefore submitted a proposal to the WHO Polio Research Committee in Geneva to study the establishment of a surveillance and response network for immunodeficiency-related vaccine-derived poliovirus in India. The proposal was aligned with the Global Polio Eradication Initiative’s draft guidelines for implementing poliovirus surveillance among patients with primary immunodeficiency disorders.^19^ The proposal was approved by the Committee in January 2019 and the study began in January 2020. In this article we describe the study protocol and discuss lessons that might be useful to other countries trying to establish immunodeficiency-related vaccine-derived poliovirus surveillance.

## Study protocol

### Study design

**Fig. 1:**
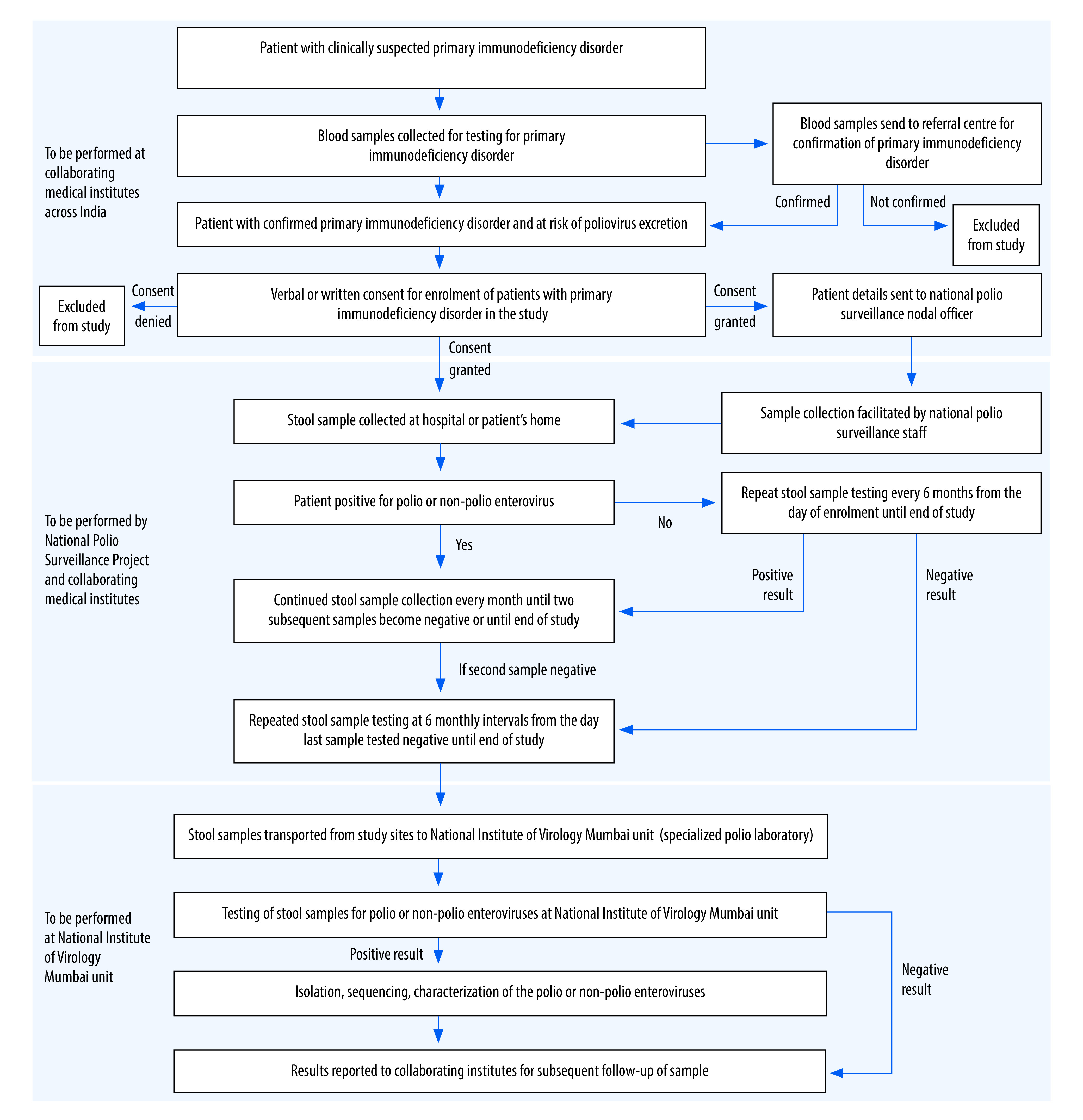
Flowchart for case enrolment, sample collection and testing in the study of poliovirus surveillance in patients with primary immunodeficiencies, India

The study was conducted from January 2020 to December 2021 at seven sites at different medical institutes in India. The initial aim was to enrol 150 patients with diagnosed primary immunodeficiency disorder. We followed up patients whose stool samples were positive for enterovirus infection, as per the study protocol (Box 1; Fig. 1). The response to detection of a patient with primary immunodeficiency and confirmed poliovirus excretion would be case investigation and community management as per the WHO and national guidelines for such an event.^20^

Box 1 **Summary of the study design for poliovirus surveillance in patients with primary immunodeficiencies, India**

***Case definition***

Patients of any age with confirmed primary immunodeficiency disorder. Primary immunodeficiency disorders are a heterogeneous group of inherited disorders due to developmental defects or dysfunction of the immune system components.

***Clinical samples***

• Stool specimens.

• Serum samples, for patients positive for immunodeficiency-related vaccine-derived poliovirus.

***Study population***

• Patients already diagnosed with primary immunodeficiency disorder, routinely visiting the primary immunodeficiency disorder outpatient department of hospitals for check-up and intravenous immunoglobulin therapy.

• Patients newly diagnosed with primary immunodeficiency disorder.

***Study period***

• Phase 1: January 2020 to December 2021.

• Phase 2: January 2022 to December 2023.

***Inclusion criteria***

• Patients confirmed with primary immunodeficiency of any age: (i) antibody disorder, including hypogammaglobulinaemia, agammaglobulinaemia, X-linked agammaglobulinaemia, and other antibody deficiencies; (ii) severe combined immunodeficiency disorder and other combined humoral and T-cell deficiencies; or (iii) others, including major histocompatibility complex deficiencies or immunodeficiency-centromeric facial-anomalies syndrome and others.

• Patients attending the study medical institute for medical care.

• Patients whose family provide consent for the study.

***Exclusion criteria***

Patients with transitory or secondary immunodeficiency; that is, related to infections including human immunodeficiency virus, chronic illness or treatment with immunosuppressive therapy.

***Data collection***

• Study site data includes case investigation details, sample collection details, number of cases enrolled and samples collected from the study sites.

• Patient data includes demographic, clinical, virological and vaccination details.

• Laboratory data includes case and sample records, test details and results, inventory and storage details.

**Data analysis**

• Data on follow-up samples will be recorded for each patient, along with the data from enrolment.

• Data will be analysed periodically for further follow-up.

• Patients positive for immunodeficiency-related vaccine-derived poliovirus will be followed up immediately as per World Health Organization guidelines.

### Ethical clearance

The study received approval from the institutional ethical committee of the National Institute of Virology and the ethics review committees of all study sites and institutes. The study protocol was also approved by the ethics review committee of WHO Headquarters in Geneva. Free and informed consent of the patient or their legal guardian was obtained before enrolment.

### Partner institutes 

The first step was to identify medical institutes with the expertise and infrastructure to confirm primary immunodeficiency disorder at the immunological and molecular level. The National Institute of Immunohaematology and Bai Jerbai Wadia Hospital for Children, who had expertise in screening for inborn errors of immunity in patients nationwide, helped to identify centres in other areas of India that were already providing diagnosis and care for primary immunodeficiency disorders. As a result, five other hospitals situated in Bangalore, Hyderabad, Kozhikode, Lucknow and Mangalore were recruited to the study, to a total of seven sites, in a collaboration with the National Institute of Virology Mumbai Unit, National Institute of Immunohaematology and WHO (Box 2). We arranged a meeting of experts and investigators to discuss and streamline the study protocol to ensure uniformity in its implementation across sites. Staff from WHO India Country Office and the National Institute of Virology Mumbai Unit, conducted site visits to assess the preparedness of the sites in terms of staffing, the required space for patient counselling, a deep freezer for sample storage, study documents and institutional support for the study.

Box 2 **Study sites in phase 1 and phase 2 of the study of poliovirus surveillance in patients with primary immunodeficiencies, India, by national polio surveillance programme region**

***Phase 1, January 2020 to December 2021***

***Uttar Pradesh***

Sanjay Gandhi Postgraduate Institute of Medical Sciences, Lucknow, Uttar Pradesh.

***Central***

Bai Jerbai Wadia Hospital for Children, Mumbai, Maharashtra.

Indian Council of Medical Research National Institute of Immunohaematology, Mumbai, Maharashtra.

***South***

Calicut Medical College, Kozhikode, Kerala.

Aster CMI, Bangalore, Karnataka.

Kasturba Medical College, Mangalore, Karnataka.

Nizam’s Institute of Medical Sciences, Hyderabad, Telangana.

***Phase 2, January 2022 to December 2023***

***North***

Post Graduate Institute of Medical Education & Research, Chandigarh.

Dr Rajendra Prasad Government Medical College, Kangra, Himachal Pradesh.

Sawai Maan Singh Medical College, Jaipur, Rajasthan.

All India Institute of Medical Sciences, Rishikesh, Uttarakhand.

Sanjay Gandhi Postgraduate Institute of Medical Sciences, Lucknow, Uttar Pradesh.

King George's Medical University, Lucknow, Uttar Pradesh.

Super Speciality Hospital, Noida, Uttar Pradesh.

***Central***

Bai Jerbai Wadia Hospital for Children, Mumbai, Maharashtra.

Indian Council of Medical Research National Institute of Immunohaematology, Mumbai, Maharashtra.

All India Institute of Medical Sciences, Bhopal, Madhya Pradesh.

***East***

Kalinga Institute of Medical Sciences, Bhubaneswar, Odisha.

All India Institute of Medical Sciences, Bhubaneswar, Odisha.

Gauhati Medical College, Guwahati, Assam.

***South***

Calicut Medical College, Kozhikode, Kerala.

Aster Cauvery Medical Institute, Bangalore, Karnataka.

Kasturba Medical College, Mangalore, Karnataka.

Nizam’s Institute of Medical Sciences, Hyderabad, Telangana.

Apollo Hospitals, Chennai, Tamil Nadu.

Institute of Child Health, Chennai, Tamil Nadu.

Jawaharlal Institute of Postgraduate Medical Education & Research, Puducherry.

Christian Medical College, Vellore, Tamil Nadu.

### Staff training 

The study staff at the selected institutes across India comprised clinicians, focal persons and other medical staff, including field staff from the WHO national polio surveillance project. We conducted a one-day training at each hospital or medical institute for staff, with the involvement of the National Institute of Virology Mumbai Unit and WHO India Country Office. The training involved procedures for counselling patients, obtaining consent for participation, enrolment of patients, collection of patients’ data on forms, collection of stool samples and follow-up of patients. WHO polio surveillance field staff and the medical staff were trained to facilitate home collection of stool samples. 

### Study coordination

The study required thorough coordination, extensive training, technical support, routine monitoring and regular follow-up between the team at the National Institute of Virology Mumbai Unit and the field sites. The National Institute of Virology Mumbai Unit was the main coordinating centre for the study and kept the records of patients enrolled and of the collection, follow-up and shipment of samples. The focal person at each study site coordinated with the Institute team about sample collection and transport issues and queries. The project was based on strong coordination among the Institute, study sites and WHO India Country Office. Since the study sites had different facilities for diagnosis and management of patients with primary immunodeficiency disorder, each site needed individual advice on patient enrolment and sample collection.

### Patient enrolment 

Although our pilot study only enrolled children, for this study we included patients of all age groups. Clinicians at the study sites (the site investigators) identified suspected cases of primary immunodeficiency disorder based on the Jeffrey Modell Foundation warning signs for the diagnosis of primary immunodeficiency.^21^ Any patient fulfilling the criteria of two or more positive signs were considered for confirmation of primary immunodeficiency disorder (Box 3). Patients eligible for enrolment in the study were (i) individuals already diagnosed with primary immunodeficiency disorder and routinely visiting the hospital or clinic for a check-up and intravenous immunoglobulin treatment; or (ii) individuals newly diagnosed with primary immunodeficiency disorder. These patients were approached for participation in the study and enrolled after they had given consent.

Box 3 **The Jeffrey Modell Foundation 10 warning signs of primary immunodeficiency when diagnosing for inborn errors of immunity**

1. Four or more new ear infections within 1 year.

2. Two or more serious sinus infections within 1 year.

3. Two or more months on antibiotics with little effect.

4. Two or more pneumonias within 1 year.

5. Failure of an infant to gain weight or grow normally.

6. Recurrent, deep skin or organ abscesses.

7. Persistent thrush in mouth or fungal infection on the skin.

8. Need for intravenous antibiotics to clear infections.

9. Two or more deep-seated infections including septicaemia.

10. A family history of primary immunodeficiency disorder.

Source: Jeffrey Modell Foundation, 1993.^21^

### Sample handling

**Fig. 2:**
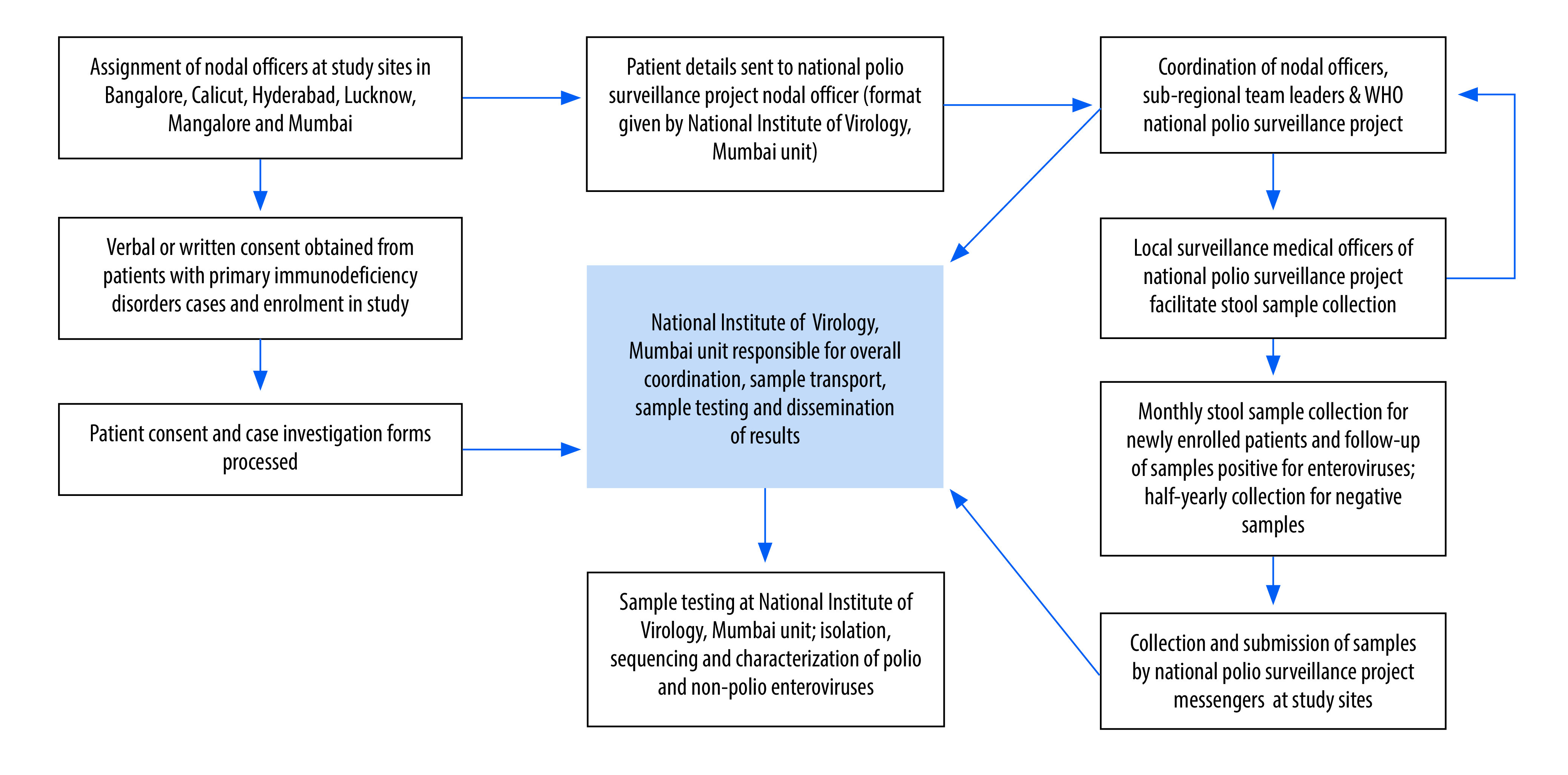
Protocol for sample collection through the national polio surveillance programme in the study of poliovirus surveillance in patients with primary immunodeficiencies, India

We planned to collect stool samples from enrolled patients either at the hospital treating them (during hospitalization or regular follow-up visits) or at their home. For home collection, the patients were provided with stool containers and a carrier with frozen ice packs to transport samples and maintain cold chain. After enrolment, patients provided two samples 1 month apart that were stored at −20 °C. All samples collected at a given site during a month, along with the related documents, were sent to the National Institute of Virology Mumbai Unit for testing for enteroviruses. At the Institute, the stool samples were anonymized and stored at −20 °C until further processing, as per WHO guidelines.^22^ The processed faecal samples were cultured for detection of viruses, with further isolation and characterization of virus isolates if samples were found to be positive (Fig. 1 and Fig. 2). Tests included are serum immunoglobulin levels, blood immunophenotyping by flow cytometry and molecular testing, up to next-generation sequencing to confirm the defect at genetic level.

WHO: World Health Organization

### Follow-up

Focal persons assigned by the site investigators followed up patients regularly to obtain monthly consecutive samples (two samples from each person) and remind patients about the subsequent samples 15 days in advance. Patients whose samples were positive for polio or non-polio enteroviruses were subsequently followed up until two subsequent samples tested negative. Patients with primary immunodeficiency who tested negative for enteroviruses in two consecutive samples were followed up at 6-monthly intervals (Fig. 1).

### Case management

The identification of a patient with primary immunodeficiency and confirmed poliovirus excretion was immediately notified to the respective site investigators and WHO India Country Office officials by the principal investigator. Key people were notified at the National Institute of Virology, WHO and the health ministry. Set guidelines were followed for counselling the patient and their close family about isolation of the patient, vaccination with inactivated poliovirus vaccine and procedures for discarding of faecal material. The results of the processed samples were provided within a month of collection to follow up the positive samples at monthly intervals.

The case and community investigation of a positive case was handled by the WHO national polio surveillance field units with the support of the Indian Council of Medical Research and local health authority, coordinated by the health ministry. An investigation of the risks of spillover of immunodeficiency-related vaccine-derived poliovirus into the community was conducted. Stool samples of select children in the local area were tested for poliovirus excretion. The serum sample of the polio-positive child was tested for polio antibody titre. The protocol states that identified polio-positive patients would continue to be clinically managed by the study site at the discretion of the treating physician. The poliovirus antiviral agent (Pocapavir, Virodefense, United States of America) along with a protease inhibitor may be considered in future for treatment of polio-positive patients, subject to Indian regulatory approval. The case management would depend on the type of poliovirus isolated, the sequencing data and the presence of risk factors for community transmission.

### Data management

Data from the case investigation forms from all the study sites were entered electronically and compiled as a single master database at the National Institute of Virology Mumbai Unit. The database comprised patients’ demographic data, clinical history, symptoms and immunization status and the results of the sample analysis. The data were analysed periodically to monitor the progress of the study and the follow-up of samples. We recorded the frequency and percentage of patients with poliovirus and non-poliovirus enterovirus by type of primary immunodeficiency disorder, site, age and sex, with further characterization in terms of nucleotide changes, if relevant. The data were shared with the collaborating study sites and WHO country office India.

### Site monitoring

We visited study sites to monitor various study-related activities. Monitoring focused on how sites managed the informed consent process with patients. Other important activities included assessing the availability of supplies at study sites, review of study-related documents and data management processes at sites. We also monitored the sample management in the study, including sample collection, storage and transport. The reasons behind loss to follow-up were also assessed to reduce patient attrition to the minimum.

## Lessons learnt

In countries using oral polio vaccine and where newborn screening is not available, as in India, it is important to screen and diagnose children with primary immunodeficiency disorder and create awareness about the consequences of oral polio vaccine in these children. However, the diagnosis of primary immunodeficiency disorder requires trained manpower and the necessary infrastructure at major hospitals to identify such cases. Therefore, working with clinicians and paediatricians is necessary to establish referral facilities in at least a few areas of the country so that the samples from more remote areas can be transferred and analysed to confirm the diagnosis. 

Parents residing in remote areas may find it inconvenient to travel to hospital sites to deposit stool samples. A system therefore needs to be developed for home collection of samples through local government health providers or existing national programmes. With the study institutes having different capacities, we faced several challenges related to collection, storage and transport of samples. Another challenge is the poor financial status of families, which deters many parents from follow-up hospital visits for expensive treatment such as bone marrow transplant after a diagnosis of primary immunodeficiency disorder in their child. 

The greatest challenge we faced was the coronavirus disease 2019 (COVID-19) pandemic which started just 4 months after the start of the study. The nation was placed under a strict lockdown for more than 2 months and most hospitals at the time were converted into COVID-19 care centres, which affected sample collection. Due to these challenges, we decided to involve staff from the national polio surveillance project. As a part of routine acute flaccid paralysis surveillance since 1997, these staff coordinate with district health officials to obtain stool samples for the polio surveillance system in India. Their networking experience across India facilitated sample collection and transport to our study sites in the districts and states or even across the border states during the COVID-19 pandemic (Fig. 2). This experience led us to further coordinate with WHO polio surveillance officials to develop standard operating procedures for sample collection. The pandemic also hampered physical visits to monitor study activities. However, we were able to assess the progress and quality of the study via periodic videoconference meetings. 

## Study outcomes

The study has provided important data on the extent of excretion of polioviruses and non-polio enteroviruses among people with primary immunodeficiency in India. We were able to analyse these outcomes in terms of age, sex, primary immunodeficiency disorder type and study site. Another important outcome was our experience with the integration of primary immunodeficiency disorder surveillance with polio surveillance in India, due to the field involvement of the WHO polio surveillance team. 

Some key achievements of the project are: (i) establishment and expansion of a network for screening long-term poliovirus excretion in patients with primary immunodeficiency disorder in India; (ii) networking of these sentinel sites with diagnostic facilities for primary immunodeficiency disorder in India; (iii) support from the national polio surveillance project in coordination with sentinel sites and government sources for collection of stool samples from the homes of patients with primary immunodeficiency disorder; (iv) integration of primary immunodeficiency disorder screening with acute flaccid paralysis surveillance in India; (v) increased awareness among paediatricians and other related clinicians about the consequences of long-term excretion of poliovirus from patients with primary immunodeficiency disorder; (vi) increased awareness within the Indian health system of the importance of avoiding oral polio vaccine in children with primary immunodeficiency disorder; (vii) development of draft national guidelines for integration of primary immunodeficiency disorder surveillance into national polio surveillance; (viii) some insights gained into the epidemiology of primary immunodeficiency disorder in India and immunodeficiency-related vaccine-derived poliovirus excretors; and (ix) development of a primary immunodeficiency disorder registry with tracking and follow-up of patients.

## Way forward

After learning from the first phase of the study, we expanded the surveillance to include additional referral hospitals in other areas of India by sensitizing clinicians to the issue, extending diagnostic testing and integrating the study with ongoing acute flaccid paralysis surveillance. The Polio Research Committee in Geneva, Switzerland, accepted this proposal of expansion to a second phase of the study which was planned to be conducted from January 2022 to December 2023. The study now has better geographical representation countrywide with the inclusion of 14 new study sites across India, to a total of 21 sites (Box 2).^23^

We plan to integrate the primary immunodeficiency disorder surveillance with the national acute flaccid paralysis surveillance to carry out long-term screening for polio and non-polioviruses in patients with primary immunodeficiencies. The immunological parameters such as cytokine responses and anti-polio antibody titres responsible for enterovirus replication and clearance of the virus will also be investigated as part of this study. The national registry of primary immunodeficiency disorders being developed by the Indian Council of Medical Research will help provide epidemiological data on people with primary immunodeficiency disorder in India. We hope that our experience will help other countries to initiate immunodeficiency-related vaccine-derived poliovirus surveillance. The aim will be to reduce the risk of polio resurgence, to inform policy-makers working on polio eradication, and to contribute information to the global surveillance database on immunodeficiency-related vaccine-derived poliovirus. 

## References

[1] Chong CY, Kam KQ, Yung CF. Combating a resurgence of poliomyelitis through public health surveillance and vaccination. Ann Acad Med Singap. 2023 Jan;52(1):17–26. 10.47102/annals-acadmedsg.2022390 36730802

[2] Detection of circulating vaccine-derived poliovirus 2 (cVDPV2) in environmental samples – the United Kingdom of Great Britain and Northern Ireland and the United States of America [internet]. Geneva: World Health Organization; 2022. Available from: https://www.who.int/emergencies/disease-outbreak-news/item/2022-DON408 [cited 2023 Feb 21].

[3] Centers for Disease Control and Prevention (CDC). Update on vaccine-derived polioviruses – worldwide, April 2011–June 2012. MMWR Morb Mortal Wkly Rep. 2012 Sep 21;61:741–6. 22992572

[4] Pliaka V, Kyriakopoulou Z, Markoulatos P. Risks associated with the use of live-attenuated vaccine poliovirus strains and the strategies for control and eradication of paralytic poliomyelitis. Expert Rev Vaccines. 2012 May;11(5):609–28. 10.1586/erv.12.28 22827246

[5] Vaccine-derived poliviruses: circulating vaccine-derived polioviruses [internet]. Geneva: Global Polio Eradication Initiative; 2013. Available from: http://www.polioeradication.org/Polioandprevention/Thevirus/Vaccinederivedpolioviruses.aspx [cited 2023 Feb 2].

[6] Macklin G, Liao Y, Takane M, Dooling K, Gilmour S, Mach O, et al.; iVDPV Working Group. Prolonged excretion of poliovirus among individuals with primary immunodeficiency disorder: an analysis of the World Health Organization registry. Front Immunol. 2017 Sep 25;8:1103. 10.3389/fimmu.2017.01103 28993765 PMC5622164

[7] Tangye SG, Al-Herz W, Bousfiha A, Chatila T, Cunningham-Rundles C, Etzioni A, et al. Human inborn errors of immunity: 2019 update on the classification from the International Union of Immunological Societies Expert Committee. [published correction appears in J Clin Immunol. 2020 Feb 22]. J Clin Immunol. 2020 Jan;40(1):24–64. 10.1007/s10875-019-00737-x 31953710 PMC7082301

[8] Tangye SG, Al-Herz W, Bousfiha A, Cunningham-Rundles C, Franco JL, Holland SM, et al. Human inborn errors of immunity: 2022 update on the classification from the International Union of Immunological Societies Expert Committee. J Clin Immunol. 2022 Oct;42(7):1473–507. 10.1007/s10875-022-01289-3 35748970 PMC9244088

[9] van Zelm MC, Condino-Neto A, Barbouche MR. Editorial: primary immunodeficiencies worldwide. Front Immunol. 2020 Jan 22;10:3148. 10.3389/fimmu.2019.03148 32038648 PMC6987400

[10] Polio this week as of 19 July 2022 [internet]. Geneva: World Health Organization; 2022. Available from: https://polioeradication.org/polio-today/polio-now/this-week/ [cited 2021 Dec 2].

[11] Jindal AK, Pilania RK, Rawat A, Singh S. Primary immunodeficiency disorders in India – a situational review. Front Immunol. 2017 Jun 19;8:714. 10.3389/fimmu.2017.00714 28674536 PMC5474457

[12] Indian Society for Primary Immune Deficiency [internet]. Chandigarh: Indian Society for Primary Immune Deficiency; 2017. Available from: https://ispid.org.in/ [cited 2021 Nov 24].

[13] Foundation for Primary Immunodeficiency Diseases [internet]. Irvine: Foundation for Primary Immunodeficiency Diseases; 2020. Available from: https://fpid.org/wp/ [cited 2021 Nov 24].

[14] Indian Patients Society for Primary Immunodeficiency [internet]. Bhubaneswar: Indian Patients Society for Primary Immunodeficiency; 2015. Available from: https://www.ipspiindia.org/ [cited 2021 Nov 24].

[15] Primary Immunodeficiency Patients’ Welfare Society [internet]. Bangalore: Primary Immunodeficiency Patients’ Welfare Society; 2020. Available from: https://www.facebook.com/PIDPWS/ [cited 2021 Nov 24].

[16] Global polio eradication action plan 2022–2024. Geneva: Global Polio Eradication Initiative; 2022. Available from: https://polioeradication.org/wp-content/uploads/2022/05/GPSAP-2022-2024-EN.pdf [cited 2021 Nov 24].

[17] Mohanty MC, Madkaikar MR, Desai M, Taur P, Nalavade UP, Sharma DK, et al. Poliovirus excretion in children with primary immunodeficiency disorders, India. Emerg Infect Dis. 2017 Oct;23(10):1664–70. 10.3201/eid2310.170724 28930011 PMC5621533

[18] Mohanty MC, Madkaikar MR, Desai M, Aluri J, Varose SY, Taur P, et al. Natural clearance of prolonged VDPV infection in a child with primary immunodeficiency disorder. Front Immunol. 2019 Jul 23;10:1567. 10.3389/fimmu.2019.01567 31396204 PMC6663979

[19] Draft guidelines for implementing poliovirus surveillance among patients with primary immunodeficiency disorders (PIDs). Geneva: Global Polio Eradication Initiative; 2019. Available from: https://polioeradication.org/wp-content/uploads/2022/06/Guidelines-for-Implementing-PID-Suveillance_EN.pdf [cited 2021 Nov 24].

[20] Guidelines for implementing poliovirus surveillance among patients with primary immunodeficiency disorders (PIDs). Geneva: Global Polio Eradication Initiative; 2022. Available from: https://polioeradication.org/wp-content/uploads/2022/06/Guidelines-for-Implementing-PID-Suveillance_EN.pdf [cited 2023 Mar 11].

[21] 10 warning signs of primary immunodeficiency [internet]. New York: Jeffrey Modell Foundation; 1993. Available from: https://info4pi.org/library/educational-materials/ [cited 2021 Nov 24].

[22] Polio laboratory manual, 4th ed. Geneva: World Health Organization; 2004. Available from: https://apps.who.int/iris/bitstream/handle/10665/68762/WHO_IVB_04.10.pdf?sequence=1&isAllowed=y [cited 2023 Mar 11].

[23] Mohanty MC, Mohammad A, Verma H, Kumar A, Madkaikar MR, Desai M, et al. Poliovirus surveillance in patients with primary immunodeficiencies, India. Supplementary files [online repository]. London: figshare; 2023. 10.6084/m9.figshare.22626874.v2 PMC10140681 37131936

